# Separation of Semiconducting Carbon Nanotubes Using Conjugated Polymer Wrapping

**DOI:** 10.3390/polym12071548

**Published:** 2020-07-13

**Authors:** Jingyi Wang, Ting Lei

**Affiliations:** Key Laboratory of Polymer Chemistry and Physics of Ministry of Education, Beijing Key Laboratory for Magnetoelectric Materials and Devices, Department of Materials Science and Engineering, College of Engineering, Peking University, Beijing 100871, China; jingyi-wang@pku.edu.cn

**Keywords:** carbon nanotubes, conjugated polymers, selective dispersion, electronic devices

## Abstract

In the past two decades, single-walled carbon nanotubes (SWNTs) have been explored for electronic applications because of their high charge carrier mobility, low-temperature solution processability and mechanical flexibility. Semiconducting SWNTs (s-SWNTs) are also considered an alternative to traditional silicon-based semiconductors. However, large-scale, as-produced SWNTs have poor solubility, and they are mixtures of metallic SWNTs (m-SWNTs) and s-SWNTs, which limits their practical applications. Conjugated polymer wrapping is a promising method to disperse and separate s-SWNTs, due to its high selectivity, high separation yield and simplicity of operation. In this review, we summarize the recent progress of the conjugated polymer wrapping method, and discuss possible separation mechanisms for s-SWNTs. We also discuss various parameters that may affect the selectivity and sorting yield. Finally, some electronic applications of polymer-sorted s-SWNTs are introduced. The aim of this review is to provide polymer chemist a basic concept of polymer based SWNT separation, as well as some polymer design strategies, influential factors and potential applications.

## 1. Introduction

Carbon nanotubes were first reported by lijima in 1991 [1]. According to the number of tube walls, carbon nanotubes (CNTs) can be divided into single-walled carbon nanotubes (SWNTs) and multi-walled carbon nanotubes (MWNTs) [2]. SWNTs have attracted great research interest, due to their unique properties, such as high carrier mobility [3], variable band gaps [4], extraordinary thermal characteristics [5], as well as the flexibility and stretchability of SWNT networks [6]. According to their different conductivities and band gaps, SWNTs can be divided into metallic SWNTs (m-SWNTs) and semiconducting SWNTs (s-SWNTs). In particular, s-SWNTs have performances comparable to or surpassing silicon-based semiconductors [7,8], which makes them suitable for a wide range of electronic applications, such as field-effect transistors (FETs) [9], photodetectors [10], solar cells [11], as well as chemical and biological sensors [12,13]. 

**Figure 1:**
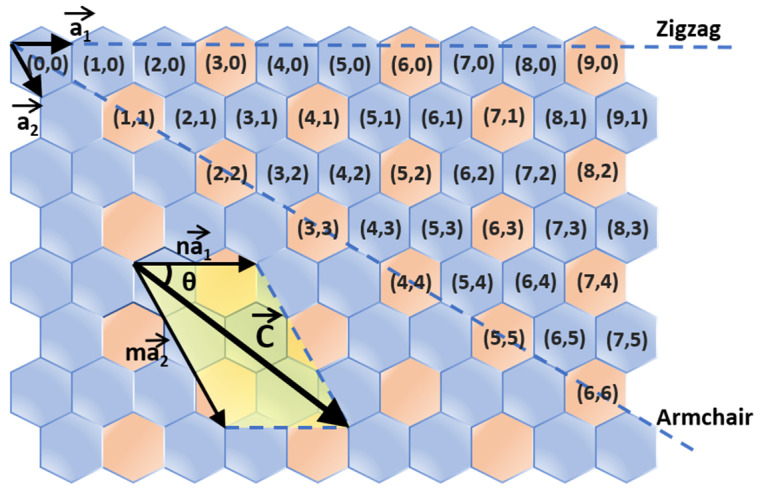
Chirality map showing the structure of single-walled carbon nanotubes (SWNTs). The chirality indices of semiconducting SWNTs (s-SWNTs) are labeled with blue, others are metallic SWNTs (m-SWNTs) (orange). Approximately 2/3 of as-produced SWNTs are semiconducting.

Essentially, the conductivity of SWNTs depends on their chiralities [14]. SWNTs can be considered a curled single-layer graphene, which rolls up along a vector ***C***, as shown in Figure 1, defined by

(1) $$C=na_{1}+ma_{2}$$

where ***a*_1_** and ***a_2_*** are a pair of basis vectors of the graphene sheet, *n* and *m* are two indices describing the chirality of the SWNTs. The chirality (*n*, *m*) determines whether the carbon nanotube is metallic or semiconducting, as well as the tube diameter (*d*) and chiral angle (*θ*). When |*n* − *m*| = 3q (q is an integer), the tube is metallic, while when |*n* − *m*| = 3q ± 1, it is semiconducting. We can also calculate the tube diameter (*d*) and chiral angle (*θ*) using the following formulas:

(2) $$d=\frac{a}{\pi }\sqrt{n^{2}+m^{2}+nm}$$

(3) $$\;\;\theta =arctan\left(\frac{\sqrt{3m}}{2n+m}\right)$$

where *a* is the length of the base vector. For s-SWNTs, the band gap is inversely proportional to the tube diameter as illustrated by the following formula [3]:

(4) $$E=\gamma (\frac{2Rc\text{-}c}{d_{CNT}})$$

where γ is the overlap integral of the nearest neighbor carbon atom (∼3 eV), *R*_C-C_ is the carbon–carbon bond length and *d_CNT_* is the tube diameter.

Several methods for the mass production of SWNTs have been developed, including arc discharge [15], laser ablation [16], plasma torch [17], high-pressure carbon monoxide (HiPCO) [18] and the decomposition of CO on Co–Mo catalysts (CoMoCAT) [19]. Different methods produce SWNTs with different diameter distributions [20]. For example, CoMoCAT SWNTs and HiPCO SWNTs have smaller diameters: 0.7–0.9 nm and 0.8–1.2 nm respectively. Plasma torch SWNTs and arc discharge SWNTs have larger diameters, 1.1–1.5 nm and 1.2–1.7 nm, respectively. The SWNT contents (mixture of m-SWNTs and s-SWNTs) of these commercially available as-produced SWNTs are about 30–70 wt% [20], and others are amorphous carbons and catalysts. For most electronic applications, only pure s-SWNTs or pure m-SWNTs are needed. For example, the channel of the FET only needs high purity s-SWNTs, the presence of m-SWNTs may cause short circuit, especially for nano-sized devices. The chemical vapor deposition (CVD) method can provide high purity s-SWNTs (~99.9%) [21], however, it is costly and cannot be produced at a large-scale, and the tube qualities are not satisfactory. In addition, the semiconducting purity is still far lower than industrial requirement for large-scale circuits (m-SWNTs should be lower than ppb level) [22]. Therefore, the CVD method is unsuitable for large-scale production and still needs further improvement. Additionally, the strong interactions between SWNTs make them easy to bundle together and cannot be solution processed, which greatly hinders their processability [23]. Therefore, many strategies have been proposed for post-processing the as-produced SWNTs for dispersing and sorting SWNTs with different metallicities, diameters or chiralities [24,25]. 

**Figure 2:**
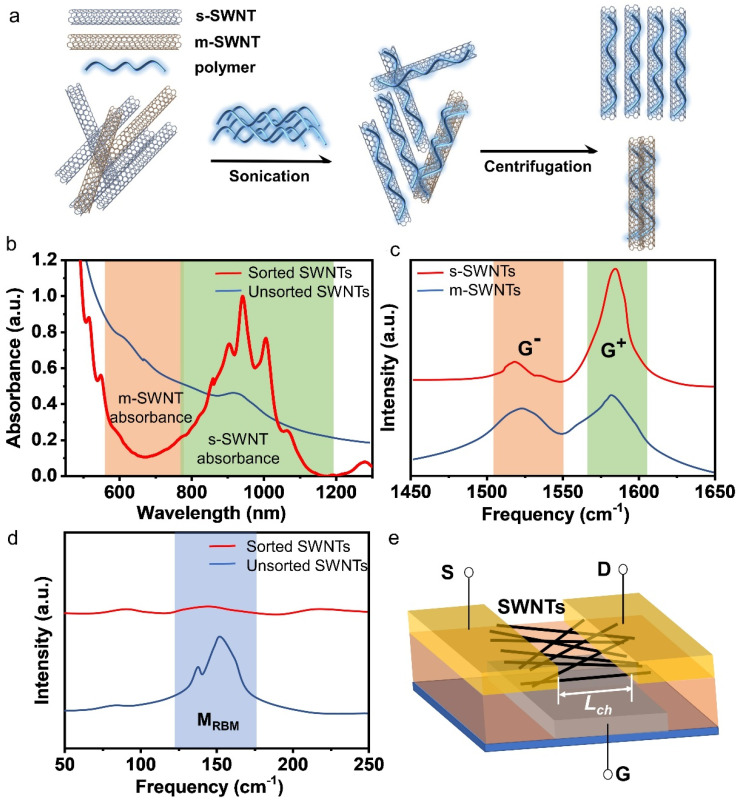
(**a**) Basic process of the conjugated polymer wrapping method for selectively dispersing s-SWNTs. During the sonication process, polymer will wrap around the SWNTs. Polymer-wrapped m-SWNTs tend to bundle together and form precipitation after centrifugation, leaving the sorted and individualized s-SWNTs in the supernatant; (**b**) UV-Vis-IR spectroscopy of sorted and unsorted SWNTs. The purity of s-SWNTs can be evaluated by comparing the absorption bands of m-SWNTs (typically between 600 nm and 800 nm for arc discharge SWNTs) and the S_22_ absorption band of s-SWNTs (typically between 800 nm and 1200 nm for arc discharge SWNTs). The absorbance of m-SWNTs significantly reduced after the sorting process; (**c**) Raman spectroscopy measurement for the G peaks of SWNTs which is split into two types: G^+^ and G^−^. G^+^ is induced by atomic displacement along the tube axis, while G^−^ is induced by the displacement along the circumferential direction of the tube. The G^−^ peak of m-SWNTs is stronger and wider than that of s-SWNTs, because m-SWNTs have more free electrons; (**d**) Raman spectroscopy for radial breathing mode (RBM) vibration peaks of SWNTs. Under certain excitation wavelengths, the signals from m-SWNTs disappear after sorting (peaks in the range of 140–190 cm^–1^ come from m-SWNTs under 785 nm excitation); (**e**) schematic diagram of short-channel transistor device for evaluating the purity of the sorted tubes. S, D and G stand for source, drain and gate electrode, respectively.

In general, post-processing methods can be divided into covalent and non-covalent methods [26,27]. The covalent methods modify and separate SWNTs through chemical reactions. It will introduce defects to the side walls of SWNTs, resulting in decreased physical properties for SWNTs. While using non-covalent methods, the sp^2^ carbon hybrid of SWNTs is retained, and the intrinsic properties of SWNTs remain unchanged. To date, widely reported non-covalent methods include density gradient ultracentrifugation [28,29], DNA wrapping [30], gel chromatography [31], electrophoresis [32], aqueous two-phase extraction [33] and conjugated polymer wrapping [34]. Among all these methods, conjugated polymer wrapping has been widely studied, because, compared to other methods, it tends to realize a preferential dispersing of s-SWNTs [35], and can achieve both high selectivity (>99%) and high yield (>10%) [36]. It only needs simple equipment, operation (sonication and centrifugation) and a short processing time, which makes it a suitable method for large-scale production [37]. In addition, through rational structural design, the conjugated polymer can recognize near mono-chiral s-SWNTs [38], which is more desired for high-performance computing [22]. The process of conjugated polymer wrapping mainly includes two steps (Figure 2a): (1) mixing raw SWNTs and conjugated polymers in an organic solvent, dispersing SWNTs with polymer wrapping by sonication; (2) settling impurities and metallic tubes by high-speed centrifugation, leaving the supernatant containing mainly s-SWNTs for subsequent analysis. The purity of s-SWNTs is usually first evaluated by UV-Vis-IR (Figure 2b) [36,39] and Raman spectroscopy (Figure 2c,d) [40]. We can also calculate the yield of s-SWNTs (the mass percentage of enriched s-SWNTs relative to total as-produced SWNT content) from UV-Vis-IR spectroscopy using Beers law (*A* = *εlc*) [36]. Photoluminescence (PL) spectroscopy is a powerful tool for detecting the chirality of s-SWNTs [41]. Moreover, when the purity of s-SWNTs is high (>99%), the electrical property measurement of a short channel FET (whose channel length should be smaller than the length of the tube, Figure 2e) is often used to obtain a direct and accurate evaluation of the purity [42]. 

In this review, we focus on the separation of SWNTs by conjugated polymer wrapping method. We first summarize conjugated polymers reported for separation and try to sort out the effect of polymer structures on the selectivity towards SWNTs with different diameters or chiralities. We also review removable/recyclable polymers for sorting since they provide cleaner SWNTs and lower polymer costs. Second, we discuss possible sorting mechanisms in terms of the separation of s-SWNTs and m-SWNTs, and possible parameters that affect the selectivity and sorting yield, such as polymer molecular weight, polymer/SWNT ratio, solvent and sonication temperature, etc. Third, we introduce several recent electronic applications of polymer-sorted SWNTs, mainly including logic/analog circuits and flexible/stretchable electronic devices. Finally, we will give an outlook for the development and future of conjugated polymer wrapping method and the sorted SWNTs.

## 2. Conjugated Polymers Used for Separation

Nish et al. reported the separation of different types of SWNTs for the first time with fluorene-based polymers, and the separated species are mainly semiconducting tubes [34]. Since then, more conjugated polymers have been explored for s-SWNT separation, including polyfluorenes, polycarbozoles, polythiophenes and donor–acceptor (D-A) polymers. To address the polymer contamination and cost issues, removable/recyclable polymers are also designed for separation. The sorting purity and yield of s-SWNTs are closely related to the polymer structure that determines the interactions between the polymer and SWNTs. Therefore, in this part, we summarize the typical polymers used for separating, and give an emphasis on the effect of the polymer structure on separation.

### 2.1. Polymer Backbone Structure Developed for Separation

**Figure 3:**
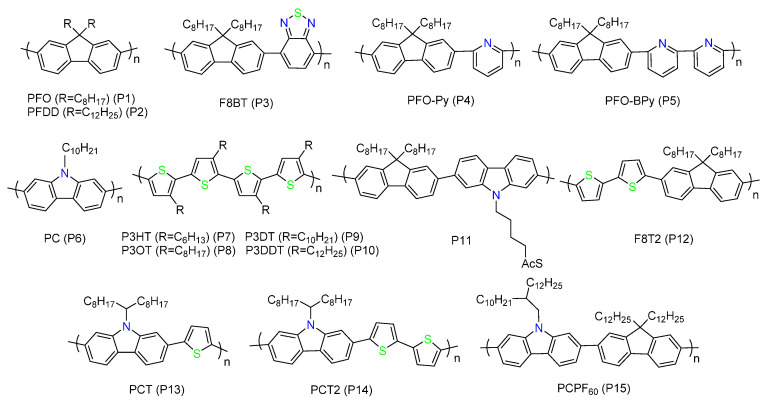
Structures of typical conjugated polymers used for sorting s-SWNTs.

So far, a variety of polymers have been developed for SWNTs separation. Some typical polymers are shown in Figure 3. Poly(9,9-dioctylfluorenyl-2,7-diyl) (PFO) (P1) is the first one reported to show good performance on separating s-SWNTs [34,43]. It has a strong selectivity towards small diameter (<1.1 nm) and high chiral angle (>24.5°) s-SWNTs [43]. The dominant selected chirality is (8, 6) for HiPCO SWNTs. It was proposed that the selectivity was due to the electronic interactions between the polymer and SWNTs, which is sensitive to the structure and electronic type of SWNTs [34]. Gao et al. reported that PFO was easy to form spiral conformation in toluene, and they speculated that it was closely related to the selectivity [44]. They calculated the area of SWNT surface wrapped by PFO and the interaction between the polymer and SWNTs. They found that the values were the largest when the polymer wrapped (8, 6) SWNT using a spiral conformation, which is consistent with the experiment result. Therefore, the structural matching of PFO and (8, 6) SWNT, as well as a suitable conformation of PFO in solvent contribute to the selectivity. 

When the SWNT diameter is greater than 1.1 nm, the dispersion ability of PFO will decrease significantly [36]. However, SWNTs with large diameters have more advantages over small-diameter SWNTs in electronic applications. For example, the maximum carrier mobility is proportional to the square of the tube diameter [45], and the use of SWNTs with large diameters in FETs can reduce the contact resistance between source/drain electrodes and SWNTs, thus increasing the current density and the on state current *I*_on_ [46]. Meanwhile, large diameter s-SWNTs have narrow band gaps and can absorb infrared solar spectrum, increasing the absorbance range, which is also useful for photovoltaic (PV) devices [36]. In addition, PFO itself has some limitations. For example, it has large band gap which prevents the photovoltaic application of the PFO/SWNT complex [47]. 

**Figure 4:**
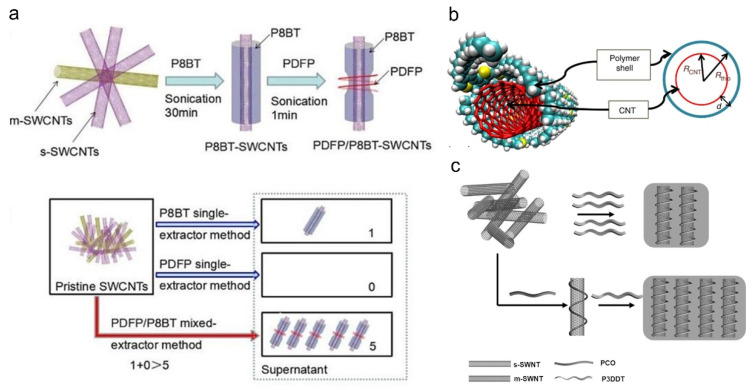
(**a**) A two-step sonication used to improve the yield of P8BT-sorted s-SWNTs and the schematic diagram of its principle. Reprinted with permission from Zhang, P. et al., 2019. Copyright 2019 John Wiley and Sons; (**b**) geometrical model of a “Polymer shell” representing the polymer/SWNT superstructure structure. R_CNT_ is the SWNT diameter, R_thio_ is the shell diameter, d is the distance between the “Polymer shell” and SWNT. Reprinted with permission from Lee, H.W. et al., 2011. Copyright 2011 Springer Nature; (**c**) the mixed-extractor strategy using P3DDT/PCO to sort s-SWNTs with a high yield. Reprinted with permission from Liu, D. et al., 2017. Copyright 2017 John Wiley and Sons.

Iijima et al. found that F8BT (P3) could enrich (15, 4) SWNTs with a diameter of 1.38 nm [48]. i.e., F8BT is selective for s-SWNTs with larger diameters, and it has smaller band gap compared with PFO [49]. The authors believed that the selectivity was derived from both the structural and the energy matching between the polymer and (15, 4) SWNT [48]. Research shows that the energy levels of F8BT are very close to the third van Hove singularities of (15, 4) SWNT. At the same time, F8BT also prefers to select s-SWNTs with medium chiral angles (*θ* ≈ 19°) [34,43]. The ability of F8BT to extract SWNTs with smaller chiral angles than PFO can be attributed to the conformation change of the polymer by introducing the benzothiadiazole (BT) monomer to backbone [38]. However, the sorting yield of s-SWNTs by F8BT is low, which needs to be improved. Recently, Zhang et al. reported a mixed extraction strategy to improve the sorting yield of F8BT [50]. They used poly(9,9-n-dihexyl-2,7-fluorene-alt-9-phenyl-3,6-carbazole) (PDFP) to enhance the selectivity of P8BT by taking a simple two-step sonication (Figure 4a). Although PDFP itself has no selectivity towards s-SWNTs, it can stabilize the polymer/SWNT complex. Using the PDFP/P8BT mixed extraction strategy, the yield of s-SWNTs increased by five times compared with the P8BT single extraction method, and the purity of s-SWNTs was more than 99%. 

Adding pyridine groups to the backbone seems to be able to disperse larger diameter SWNTs, such as those produced by laser vaporization or arc discharge [36,51]. The extracted s-SWNTs have both high purity and high yield, with a reduced diameter abundance, which is more desired for electrical and optical uses [52,53,54,55,56]. Tange et al. found that PFO-Py (P4) could selectively extract (13, 5) SWNT [52]. The diameter of (13, 5) SWNT is 1.26 nm, with an emission wavelength near 1500 nm, which is in the third telecommunications window and necessary for near-infrared photonic devices. Molecular mechanics simulations showed that the polymer backbone of PFO-Py is wavelike, and the amplitude of the wavy chain matches the diameter of (13, 5) SWNT, which explains the selectivity [57]. Ozawa et al. reported the selective extraction of (6, 5) SWNTs using PFO-BPy (P5) [58]. The diameter of the sorted s-SWNTs was small (0.95 nm for (6, 5) s-SWNTs), because they used CoMoCAT SWNTs as raw materials. However, it achieved near mono-chiral extraction with an enrichment rate of 96–97%. Later Mistry et al. used PFO-BPy to sort laser vaporization SWNTs and obtained a high yield (>33%) with a high purity (>99%) [36]. Brady et al. reported that PFO-BPy could separate arc discharge SWNTs, and demonstrated that the s-SWNT purity was higher than 99.9% through electrical measurement [53]. Therefore, PFO-BPy shows great potential to separate SWNTs, and the sorted SWNTs are widely used for further study in high performance electronic devices [53,54,55].

Polycarbazole has similar structure with polyfluorene with a sp^2^ hybrid nitrogen atom in the central aromatic ring. It has only one side chain, which reduced the steric hindrance compared with the fluorene unit [59]. Preliminary studies on poly(N-decyl-2,7-carbazole) (P6) showed that it could selectively disperse s-SWNTs with chirality (*n* − *m*) ≥ 2 in toluene, which is complementary to polyfluorenes, which mainly disperse s-SWNTs with chirality (*n* − *m*) ≤ 2 [60]. The diameter of the sorted s-SWNTs by P6 was between 0.8 and 1.1 nm, similar to that of PFO. The molecular dynamics simulation showed different π-π interactions between the polymer and SWNTs caused a different binding energy for the polymer/SWNT complexes. For the carbazole decamer, the complex formed with (10, 2) SWNT had lower binding energy than that formed with (7, 6) SWNT, while the fluorene decamer performed the opposite. Eventually, high binding energy complexes would be selected. However, the reporters had to use density gradient ultracentrifuge to purify s-SWNTs, rather than simple centrifugation, and other report showed that polycarbazole suffered poor solubility, and had much lower dispersion efficiency than polyfluorene [61]. People usually adjust its side chain structure to overcome those drawbacks, which we will discuss in detail in the “side chain design” section. 

Previous studies showed that polythiophenes could disperse high concentration SWNTs [62,63,64], while the polymers did not exhibit selectivity. In 2011, Bao et al. found that regioregular polythiophenes (rr-P3ATs) (P7–P10) could be used for separating HiPCO SWNTs [65]. In particular, they obtained high purity and high yield s-SWNTs using regioregular poly(3-dodecylthiophene) (rr-P3DDT) (P10), which was demonstrated by spectroscopy and electrical measurement. The sorted SWNTs could be directly used to fabricate electronic devices without removing excess polymers, because P3AT itself is a high-performance semiconductor. They proposed a model of “polymer shell” (the form of highly ordered polymer/SWNT supramolecular structure) (Figure 4b) to explain the diameter and chirality selectivity, and attributed the s-SWNT preference to different polarity of s-SWNTs and m-SWNTs. The high polarity of m-SWNTs caused strong charge transfer between m-SWNTs and the polymer, therefore preventing the formation of the supramolecular structure, which was necessary for valid wrapping. Later, they used P3DDT to sort CoMoCAT SWNTs with smaller diameters (~0.76 nm) and obtained a higher yield (~25%) than before (20%) [66]. Cui et al. reported that P3DDT could also extract large-diameter SWNTs produced by arc discharge with a high purity, and they fabricated printed inverters with a maximum gain of 112 at a low voltage (−5V) with the sorted-SWNTs [67]. Li et al. developed a mixed-extractor strategy using P3DDT and poly[9-(1-octylnonyl)-9H-carbazole-2,7-diyl] (PCO) to selectively disperse SWNTs with a high yield (Figure 4c) [68]. Unlike the mixed extraction strategy used by Zhang et al., they sorted SWNTs using the mixed polymer solution with one sonication step. In this method, they used PCO as the enhancer that could disperse SWNTs first, and then the polymer was replaced by P3DDT during sonication, therefore enhancing the sorting efficiency of P3DDT. They proposed that the replacement was due to different binding ability with SWNTs of the two polymers, and demonstrated that this method was also suitable for other mixed-extractor systems, such as P3DDT/PFO.

Copolymers formed by fluorene, carbazole, and thiophene units also show good selectivity towards s-SWNTs [69,70,71,72,73]. Ozawa et al. synthesized a fluorene-carbazole copolymer (P11) and found that it can select (7, 6) and (8, 6) SWNTs [69]. They further fabricated metal-nanoparticle/SWNT hybrids by attaching gold or silver nanoparticles to SWNTs through the coordination chemistry. Cui et al. found (9,9-dioctylfluorene-co-dithiophene) (F8T2) (P12) could extract large-diameter s-SWNTs synthesized by arc discharge in toluene, *m*-xylene and xylene (a mixture of *p*-xylene, *o*-xylene and *m*-xylene). Nevertheless, the obtained yield was relativity low (~5%), and the tubes were likely to bundle together easily [70]. Li et al. synthesized two copolymers containing the carbazole and thiophene units, PCT (P13) and PCT2 (P14), with structures similar to F8T2 [71]. They were used to selectively disperse s-SWNTs and compared with F8T2. The sorted solution by PCT showed a higher concentration and seemed more stable than that sorted by PCT2 and F8T2, which might be due to the larger proportion of side chains in PCT. Recently, Rice et al. developed a copolymer named PCPF_60_ (P15) for sorting SWNTs with large diameter (>1 nm). This novel polymer selected s-SWNTs with a purity greater than 99%, which was confirmed by UV-Vis-IR and Raman spectrum [73].

**Figure 5:**
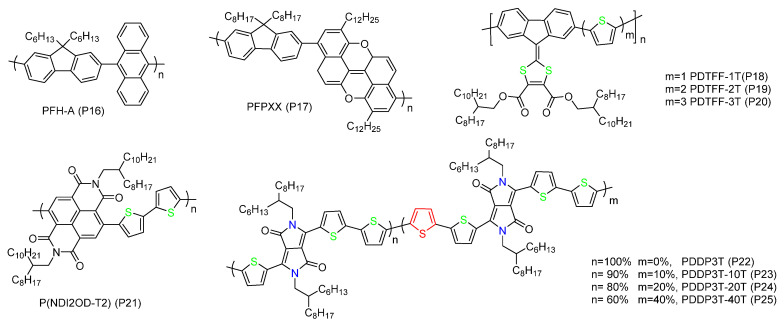
Chemical structure of copolymers containing large backbone units and some donor-acceptor (D-A) type conjugated polymers.

Some studies found that copolymers containing units with large conjugated plane could selectively disperse large-diameter SWNTs (Figure 5) [74,75,76,77,78]. Mistry et al. used PFH-A (P16) to separate laser vaporization SWNTs synthesized at different temperatures, they found that PFH-A could select near-armchair s-SWNTs over a large diameter range [44]. Later study showed that PFH-A preferred to select s-SWNTs with chiral angles larger than 23° and diameters between 1.10 and 1.31 nm [79]. Cui et al. designed an alternate copolymer PFPXX (P17) for separating SWNTs [75]. The result showed that PFPXX disperse HiPCO SWNTs well in toluene, and it could purify large diameter s-SWNTs with a high yield. They thought that the large planar aromatic ring in PFPXX greatly enhanced the π-π stacking between the polymer and SWNTs. Meanwhile, the fluorene parts with two long side chains could provide good dispersion ability for the polymer/SWNT complexes in toluene, thus raising the yield. Bao et al. reported the enrichment of s-SWNTs with a diameter range of 1.1–1.8 nm using dithiofulvalene/thiophene copolymers (P18-P20) [76]. They could obtain a stable dispersion with a high SWNT concentration. By increasing the ratio of thiophene units, the purity of s-SWNTs with small diameter increased while the yield decreased. It was related to the more flexible backbone, which could adopt a conformation adapting to the larger curvature of small-diameter SWNTs after adding more thiophene monomers. The reduction of sorting yield could attribute to fewer dithiofulvalene parts with solubilized branched side chains.

Donor–acceptor (D-A) conjugated polymers are known as the third-generation organic semiconductors, which consist of alternating electron-rich and electron-poor conjugated units. They have narrow band gaps and high charge carrier mobilities [80], suitable for electronic applications of polymer/SWNT hybrid materials [77,81]. They have been demonstrated to form strong π-π interactions with SWNTs and selectively disperse large diameter s-SWNTs [82,83,84]. Bao et al. employed a n-type D-A polymer named P(NDI2OD-T2) (P21), and found it could selectively disperse larger diameter s-SWNTs than P3DDT [82]. Molecular dynamics simulations showed when different types of SWNTs are wrapped, P(NDI2OD-T2) could perform different geometric structures, and its backbone tended to follow the hexagonal path of SWNTs and formed better π-π stacking. However, the structure of P3DDT showed little difference when wrapping SWNTs with different chirality. Later, their group reported diketopyrrolopyrrole (DPP)-based D-A polymers (P22-P25) for sorting SWNTs [84]. Through rational backbone structure design, the polymer could selectively disperse s-SWNTs with diameters between 1.4–1.6 nm, and the purity reached 99.6%. The authors found that, by incorporating more thiophene units to the backbone, both the selectivity and sorting yield for s-SWNTs could be increased. They proposed that, after adding more thiophene units, the polymer would perform stronger interaction with SWNTs. The stronger interaction resulted in an increased charge transfer between m-SWNTs and the polymers, leading to a higher selectivity. The simultaneous high yield may be due to an enhanced dispersion ability caused by a better solubility of the DPP polymer.

**Figure 6:**
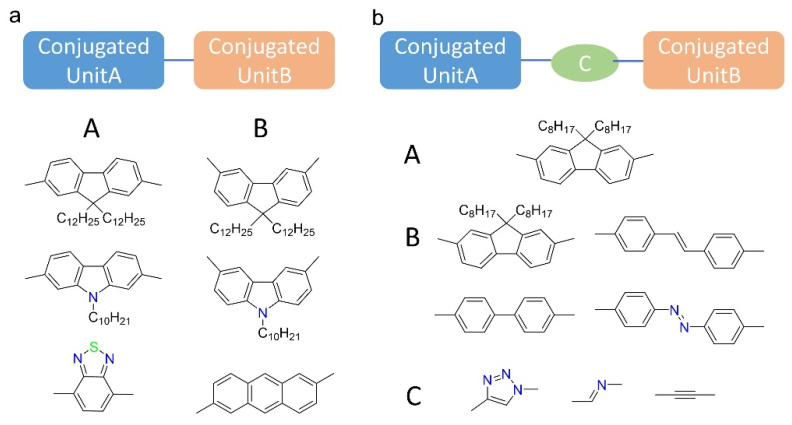
Polymer libraries established to study the effect of backbone structures on the dispersion selectivity. (**a**) Schematic diagram for partial copolymers with different monomer attachment sites. Those contain (9,9-dialkyl-2,7-fluorene) have better conjugate effect and dispersing selectivity; (**b**) polymer library of 9,9-dioctylfluorene derivatives employing biphenyle, stilbene, azobenzene and 1,2,3-triazole, azomethine, ethynyle units.

In order to understand the relationship between polymer backbone structure and selectivity, Mayor et al. established a “polymer library” to systematically study the effect of the backbone structure on the dispersion selectivity (Figure 6a) [61]. They found that polymers containing (9,9-dialkyl-3,6-fluorene) units had poor dispersing and selecting properties compared with polymers containing (9,9-dialkyl-2,7-fluorene), which has a better conjugate effect. They introduced aromatic groups to the polymer backbone of poly(2,7-fluorene) with different numbers of aromatic rings and connection sites, they obtained the best result when introducing 1, 5-linked anthracene unit to the backbone, and the polymer could select SWNT with diameter larger than 0.95 nm when HiPCO tubes were used. Different connection sites caused different polymer conformation and conjugate effect, which affects the selectivity. Later, Gerstel et al. found that, when inserting ether-bridges to the backbone of polyfluorene, the copolymers showed little selectivity or even no dispersing ability [26]. It proves again that conjugated backbone is necessary. There is one study showing that complete polymer conjugation is not needed for sorting SWNTs [85], however, conjugated monomers in backbone are necessary for the selectivity. Additionally, rigid polymers with more aromatic monomers tend to have stronger selectivity, because they can form fewer conformations, which is only suitable for wrapping SWNTs with limited chirality, and the stronger π-π interaction between aromatic units and SWNTs usually exhibits a preferred stacking path [86]. However, the dispersion ability of too rigid polymers will be poor. Gerstel et al. synthesized 14 poly(9,9-dioctylfluorene) derivatives, and several of them contained acetylene unit (Figure 6b) [87]. They found that too rigid an acetylene connection would make the polymer difficult to wrap around SWNTs, and the association between the polymer and SWNTs would be too weak. Therefore, those polymers performed no dispersion ability.

### 2.2. Polymer Side Chain Design for Separation

**Figure 7:**
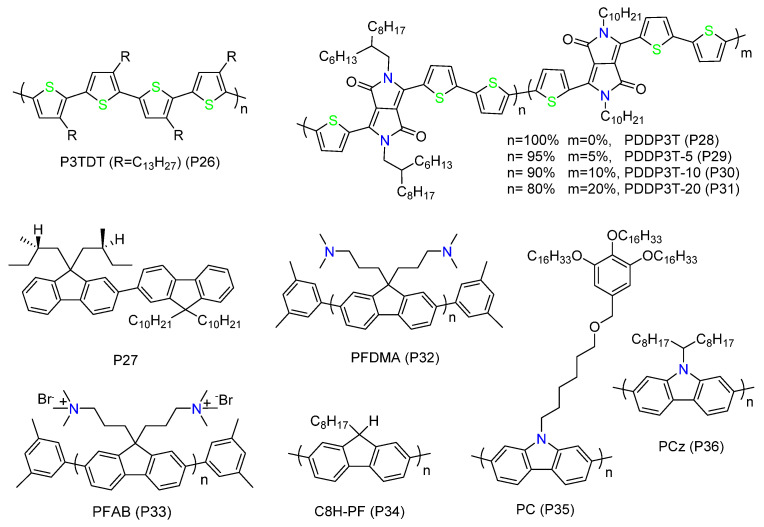
Conjugated polymers used for exploring the side chain effects on sorting.

Polymer side chains play an important role in adjusting the solubility of the polymer and altering its interaction with the SWNTs as well as with the solvent [82]. The side chain effects on sorting have been studied in many polymer systems (Figure 7) [65,88,89,90,91].

Bao et al. studied the sorting properties of rr-P3ATs (P7-P10) (P26), and they found that, by increasing the side chain length, the sorting yield of s-SWNTs would increase [65,66]. They obtained a high yield of 31% when using rr-P3TDT (P26) to disperse CoMoCAT SWNTs in toluene [66]. Molecular dynamics simulations showed that as the side chain length becomes longer, the contact area of the polymer and SWNTs would be larger. Loi et al. found similar results when studying the effect of alkyl chain length of polyfluorenes (PFs) on sorting [89]. They increased the alkyl chain length from C6 to C18, and found that polymers with longer alkyl chains could disperse larger diameter s-SWNTs (>1.2 nm) and the dispersing solution had a higher concentration. They conducted a molecular dynamics simulation and found that, except for the larger coverage area, the binding energy between the polymer and SWNTs also increased as lengthening the side chains. However, the selectivity towards s-SWNTs with specific diameters or chiralities decreases, due to more conformations the polymers form with longer alkyl chains in solvent. Especially, PF with C12 side chain (PFDD, P2) showed the highest photoluminescence yield compared to other polymers. Meanwhile, the SWNTs dispersion produced by polymers with too long side chains will have higher viscosity, which is bad for subsequent filtration (for removing excess polymers) [39]. Therefore, PFDD is usually chosen for detailed enrichment research [92,93,94]. For example, Ding et al. found that PFDD could extract near-armchair s-SWNTs with a diameter range of 1.2–1.4 nm [39]. The spectrum showed the purity of s-SWNTs was greater than 99%. Later they used the silica gel to selectively absorb the remaining m-SWNTs after PFDD sorting, which they called hybrid-conjugated polymer extraction (h-CPE) [93]. Finally, the purity was as high as 99.9%, and the yield had a 5-fold- increase compared with PFDD sorting alone.

The effect of relative content of linear side chains on separation properties was also investigated [38,83]. Ozawa et al. prepared fluorene-based copolymers containing a short, bulky chiral side chain and a long, alkyl achiral side chain (P27) [38]. They achieved selectively recognition of s-SWNTs with specific chirality by altering the ratio of chiral to achiral side chains. They proposed that introducing different amounts of bulky chiral chains would produce different steric hindrance effects for the wrapping process, leading to changes in selectivity. Bao et al. studied the effect of linear and branched side chains on the selectivity and sorting yield of D-A copolymers (P28–P31) [83]. Their study showed that D-A polymers with linear alkyl side chains have stronger van der Waals interactions with SWNTs [89]. The authors proposed that the linear alkyl chains would promote the selectivity, while the branched side chains would enhance the solubility. Finally, the polymer PDPP3T-10 (P30), which had 10% linear alkyl side chains, exhibited the highest dispersing ability and the best selectivity.

Polyfluorenes with polar side chains or asymmetric side chains can also selectively dispersing SWNTs [88,90,95]. Loi et al. explored the effect of polar side chains on the separation properties of polyfluorenes using two polymers with amine groups, named poly(9,9-di-*N*,*N*-dimethylaminopropylfluorenyl-2,7-diyl) (PFDMA) (P32) and [(*N,N,N*-trimethylammonium)-propyl]-2,7-fluorene dibromide) (PFAB) (P33) [88]. Their study found that PFDMA and PFAB could disperse larger diameter and higher concentration s-SWNTs than PFO, while the selectivity to specific SWNTs was reduced. The authors proposed that it was due to the stronger affinity of amino groups to SWNTs. In addition, absorption and PL spectrum results showed that m-SWNTs existed, as well as bundles in the dispersion of PFDMA/SWNT or PFAB/SWNT, which may be caused by the much lower molecular weight of the polymer (PFDMA: *M*_n_ = 2117 and *M*_w_ = 2704). Fukumaru et al. synthesized C8H-PF (P34) carrying a mono-alkyl chain [90]. They found that the polymer had a similar selectivity with PFO towards HiPCO SWNTs and the purity of s-SWNTs reached 99%. They thought that polyfluorene with a single long chain is enough for sorting: the interaction with SWNTs requires only one long chain. It is important for exploring the mechanism of sorting SWNTs using polyfluorenes, because it was previously thought that double long alkyl chains are necessary for the recognition of s-SWNTs [34].

In the section of the “polymer backbone structure”, we know polycarbozoles can be used for sorting s-SWNTs, while they suffer the problem of low molecular weight and poor solubility [96]. Fortunately, they are highly stable and easily derived through the reaction with the central nitrogen atom [97,98]. Therefore, various polycarbazole derivatives with adjustable side chains are prepared for separating or functionalizing SWNTs [96,99,100]. Rice et al. introduced a 3,4,5-tris(hexadecyloxy)phenyl moiety to poly(2,7-carbazole) and prepared the polymer with high molecular weight and high solubility (P35) [99]. This polymer could enrich small diameter SWNTs (≤1.15 nm) in both toluene and THF. Raman spectroscopy and PL mapping showed that the polymer disperses SWNTs well. Later, they investigated the selectivity of the polymer towards different types of SWNTs, including HiPCO and CoMoCAT SWNTs [96]. They further confirmed that the polymer preferentially interacted with s-SWNTs. Li et al. synthesized poly[9-(1-octylonoyl)-9H-carbazole-2,7-diyl] (PCz) (P36) by adding two long alkyl chains to the carbozole unit [100]. The two long side chains and the N-C bonding make the polymer more flexible and increase its solubility. The polymer could extract s-SWNTs with larger diameters (1.4~1.6 nm) and the sorted solution was stable at a high concentration. Spectral and electronic measurements estimated that the purity of s-SWNTs was up to 99.9%.

As discussed above, we can adjust the selectivity and dispersion ability of the polymer by changing the lengths and the types (e.g., linear vs. branched) of the side chain, or by introducing functional side chains. In general, a longer side chain or a larger proportion of long branched side chains will improve the solubility of the polymer and bring about more polymer wrapping conformations. More conformations lead to more area of SWNT walls being covered and, thence, change its dispersion selectivity. In particular, polymers with longer alkyl side chains exhibit stronger solubility and selectivity for large diameter s-SWNTs. Additionally, by introducing chiral side chains, we could achieve the enrichment of SWNTs with specific chirality.

### 2.3. Removable/Recyclable Polymers

**Figure 8:**
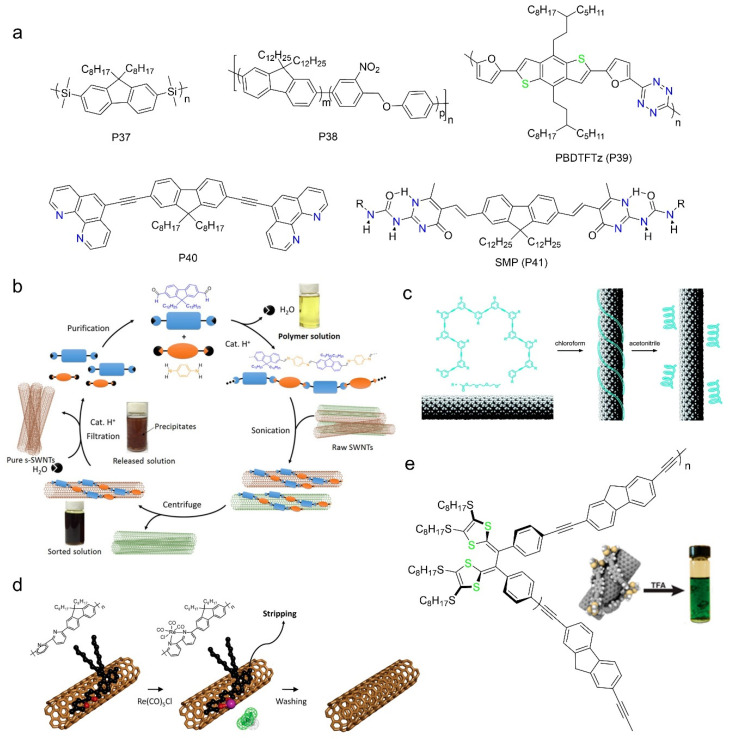
Removable/recyclable polymers used for sorting. (**a**) Chemical structures of removable/recyclable polymers containing degradable groups; (**b**) schematic diagram of PF-PD sorting and recycling process. The polymer can be decomposed by adding catalyst amount of TFA and the monomers are reused for next sorting process. Reprinted with permission from Lei, T. et al., 2016. Copyright 2016 American Chemical Society; (**c**) conformational change of foldable polymer by changing solvent. Reprinted with permission from Zhang, Z. et al., 2010. Copyright 2010 American Chemical Society; (**d**) the removal of PFO-BPy (P5) by complexation reaction with metal ions. Reprinted with permission from Joo, Y. et al., 2015. Copyright 2015 American Chemical Society; (**e**) Chemical structure of tetrathiafulvalene vinylogue (TTFV)-fluorene copolymer. Picture next to it indicates the release of the polymer from SWNTs after adding TFA. Reprinted with permission from Liang, S. et al., 2013. Copyright 2013 American Chemical Society.

The as-sorted SWNT solution by polymer wrapping usually contains a lot of excess polymers, and the electronic devices fabricated by the as-sorted SWNT solution may exhibit poor electrical performance, due to polymer contamination [101]. Therefore, several polymer removal methods have been developed recently, such as ultracentrifugation, repeated filtering and washing [102,103]. Recently, Bao et al. reported a “flocculation” method, which effectively removes many of the excess polymers [104]. However, although the free polymers in solution can be completely removed, those wrapped on the surface of SWNTs are difficult to be washed off using the above methods, due to the strong interaction between those polymers and SWNTs. The remaining polymers will still adversely affect the performances of s-SWNTs. For example, they will increase the resistance of the SWNTs network and hinder the charge transport between the SWNTs. Additionally, the cost of polymers can be really high [20]. Therefore, several items in the literature are interested in polymers that can be removed from the SWNTs and even recycled after the sorting process (Figure 8). In general, these polymers contain degradable groups or can undergo polymer conformational changes, which could then desorb from SWNTs and being removed.

#### 2.3.1. Removable Polymers Containing Degradable Functional Groups

Depolymerizing the polymers is an effective way to remove the bounded polymers. This kind of polymers usually have “reaction units” on the polymer backbones, and the depolymerization could be initiated by acid, light irradiation or heating. Wang et al. designed an alternating copolymers (P37) containing fluorene and hydrofluoric acid (HF) degradable disilane units [105]. It achieved the separation of s-SWNTs with large diameter (1.03–1.17 nm) and high chirality angle (25°–28°). The polymers wrapped on SWNTs could be easily depolymerized in HF, due to the breakage of the disilane bond. Mayor et al. reported a kind of photocleavable copolymers containing o-nitrobenzylether moiety (P38), which is photodegradable [106]. The polymer can be decomposed after irradiated by xenon lamp for a few minutes. By increasing the amounts of photodegradable units, the selectivity of the copolymer decreased while the release rate became faster upon irradiation. Recently, Malenfant et al. synthesized a decomposable s-tetrazine copolymer (PBDTFTz, P39) containing polyaromatic dithiophene fused benzene and electron-deficient s-tetrazine units [107]. This polymer exhibits no selectivity when dispersing large diameter SWNTs (~1.3 nm), due to a strong interaction with all types of nanotubes. Fortunately, it can replace PFDD (polymer used for initially s-SWNT enrichment) by a simple bath sonication process after adding it to PFDD/SWNT dispersion. The polymer can then be decomposed under UV irradiation or temperature above 250 °C. In particular, the removal process can be performed in a solid film of polymer/SWNT complex. The authors found that the contact resistance of SWNT-based transistors reduced from 0.96 kΩ to 0.24 kΩ after removing the coated polymers. However, these decomposable polymers cannot be recycled after breaking down.

Decomposable and recyclable polymers based on metal-coordination, hydrogen-bonding and imine-bonding have been reported [101,108,109,110]. Toshimitsu et al. reported coordination polymers composed of fluorene and metal complexes (P40) [108]. The coordination bond will break down when exposed to protonic acid, resulting in the depolymerization and removal of polymers. The monomers can be reused after base treatment. Bao et al. proposed a hydrogen-bonding based supramolecular polymer (P41) for purifying s-SWNTs. They could decompose and remove the polymers by adding acid to the sorted solution [109]. The polymers will form precipitation with the help of MeOH and then be collected and recycled. It could separate arc discharge s-SWNTs in toluene and achieve a high purity with a nearly 7-fold higher yield than PFDD sorting under similar conditions. However, this kind of supramolecular is difficult to synthesize. Polymers containing imine bonds can be synthesized simply and environmentally friendly, and the imine bonds break easily under a small amount of acid. The Bao group reported a removable and recyclable polymer poly[(9,9-di-n-dodecyl-2,7-fluorendiyl-dimethine)-(1,4-phenylene-dinitrilomethine)] (PF-PD) based on imine-bonding (Figure 8b) [110]. This polymer exhibited strong selectivity towards large diameter s-SWNTs. The purity of the sorted s-SWNTs was 99.7%, which was later improved to 99.997% [42], as evaluated by electrical measurement, and the yield was up to 23.7%. The PF-PD exhibits better selectivity when using low cost and low purity raw materials, greatly reducing the sorting cost. Arnold et al. synthesized poly[(9,9-di-n-octyl-2,7-fluoren-dinitrilomethine)-alt-co-(6,6′-2,2′-bipyridyl-dimethine)] (PFO-N-BPy) by introducing imine bonds to the backbone of PFO-BPy [101]. The polymer shows similar selectivity with PFO-BPy, and it can disperse SWNTs at a much lower molecular weight (~10 units). They used short channel field effect transistors to confirm that the semiconducting purity might be higher than 99.9%. Similar to PF-PD, this polymer can also be degraded to monomers to reduce the contamination of polymers in electronic devices.

#### 2.3.2. Removable/Recyclable Polymers with Conformation Change Mechanism

Zang et al. reported foldable oligomers named oligo(m-phenylene ethynylene)s to reversibly disperse and release SWNTs (Figure 8c) [111]. The polymer will change its conformation in different solvents. The conformation depends on the π-π interactions between nonadjacent monomers, as well as the solvophobic effect between the hydrophobic backbone and the solvent. It can adopt a folded, helical conformation in polar solvents other than chlorinated solvents [112]. They successfully dispersed s-SWNTs in chloroform, and then released the s-SWNTs by changing the solvent to polar acetonitrile. Copolymers containing tetrathiafulvalene vinylogue (TTFV) units can switch conformation reversibly upon oxidation/reduction [113]. Liang et al. synthesized TTFV-fluorene copolymer and demonstrated that it had similar selectivity with polyfluorenes (Figure 8e) [114]. After adding two drops of trifluoroacetic acid (TFA) to the sorting solution, the polymer changed its conformation and unwrapped SWNTs, leading to the release and precipitation of SWNTs. The polymer could recover its origin state with the help of NaHCO_3_ and be reused to sort SWNTs again. The conformation change can also be stimulated by complexation reaction with metal ions. Gopalan et al. used pentacarbonylrhenium chloride (Re(CO)_5_Cl) to trigger a drastic conformational change for PFO-BPy through chelation chemistry (Figure 8d) [115], resulting in the unwrapping and removal of the polymer. The rigidity of the PFO-BPy backbone will change upon complexation, therefore destroying the *π–π* interaction between the polymer and SWNTs. As mentioned above, PFO-BPy has both high selectivity and high yield for sorting SWNTs. This method could release up to 85% PFO-BPy from the sorted arc discharge SWNTs (*d* = 1.3–1.7 nm) and 72% from sorted CoMoCAT SWNTs (*d* = 0.7–0.8 nm).

## 3. Parameters that Affect the Selectivity and Sorting Yield

### 3.1. Possible Mechanisms for Selecting s-SWNT

In order to obtain a high selectivity to s-SWNTs or SWNTs with specific chiralities, polymers with different structures and several sorting parameters have been explored. A possible sorting mechanism that is widely accepted is based on different polarizabilities of s-SWNTs and m-SWNTs. m-SWNTs have much higher charge carrier density, and they exhibit 10^3^ times larger polarizability than s-SWNTs [116]. Therefore, there exists a strong charge transfer between m-SWNTs and conjugated polymers, and the polymer/m-SWNT complexes tend to aggregate because of the dipole-dipole interactions between them. The aggregates can settle to sediment during centrifugation, leaving the dispersed s-SWNTs in the supernatant. This mechanism can be used to explain many experiment results. For example, adding large conjugated units to the backbone helps to increase the selectivity [75,77]. Polymers with large conjugated units have stronger interactions with SWNTs, leading to an enhanced charge transfer between polymers and m-SWNTs and, thus, an increased selectivity. In addition, we usually use non-polar solvents for sorting, such as toluene and xylene [86,116]. Polar solvents such as tetrahydrofuran (THF) or dimethylformamide (DMF) etc. will enhance the solubility of the polar polymer/m-SWNT complexes, which leads to reduced selectivity.

**Figure 9:**
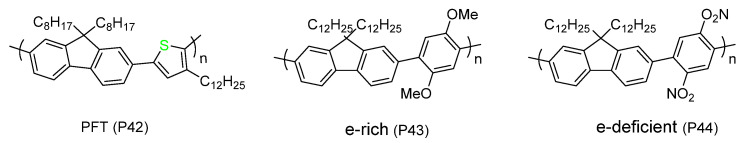
The polymers used for exploring the effect of electronic structure on selectivity.

However, some polymers can exhibit good selectivity in polar solvents THF, such as polycarbazoles (P35, P36) [96,117], which seems to run counter to the above mechanism. Several studies have found that the electronic structure of the polymer plays an important role in the selectivity (Figure 9) [118,119,120,121]. Adronov et al. found that PFT (P42), which is relatively rich in electrons, exhibited stronger supramolecular complexation with SWNTs [122]. Spectral results showed the PFT/SWNT complex had a large π-π stacking interaction, making the *π* electrons delocalized on the surface of SWNTs. Later, they found that electron-rich polymers tended to purify s-SWNTs, while the electron-deficient polymers tended to enrich m-SWNTs [123,124,125]. They adjusted the substitution groups from an electron-rich comonomer (p-dimethoxyphenyl) (P43) to an electron-deficient comonomer (p-dinitrophenyl) (P44), and successfully achieved a transition from dispersing s-SWNTs to only dispersing a mixture containing a large amount of m-SWNTs [123]. However, they cannot disperse m-SWNTs only, due to the existence of electron-rich fluorene units. Recently, the Adronov group also synthesized electron-rich carbazole-co-fluorene derivative and electron-deficient methylated fluorene-co-pyridine polymer [125]. They found that the electron-deficient polymer has a strong ability to disperse and enrich m-SWNTs, particularly after removing most of the s-SWNTs in raw materials. Therefore, they proposed that the mechanism based on the polarizability is focus on the selectively aggregation of m-SWNTs after dispersing, and they proposed a mechanism focus on the selectively exfoliation of the polymer towards different types of SWNTs during dispersion [59]. These two mechanisms may not be contradictory, and may jointly determine the selectivity [39].

Although there is not a complete theory about the mechanism, the main factors affecting the selectivity are widely believed to be van der Waals interactions (including π-π stacking) between the polymers and SWNTs. We have discussed the effects of polymer structures in the second part. Except for the polymer structure, many other parameters, such as the molecular weight, solution concentration, polymer to SWNTs ratio, solvent and temperature, will also make a significant impact on both selectivity and yield. Next, we will review related studies, hoping to provide guidance for optimizing the sorting conditions.

### 3.2. Polymer Weight

**Figure 10:**
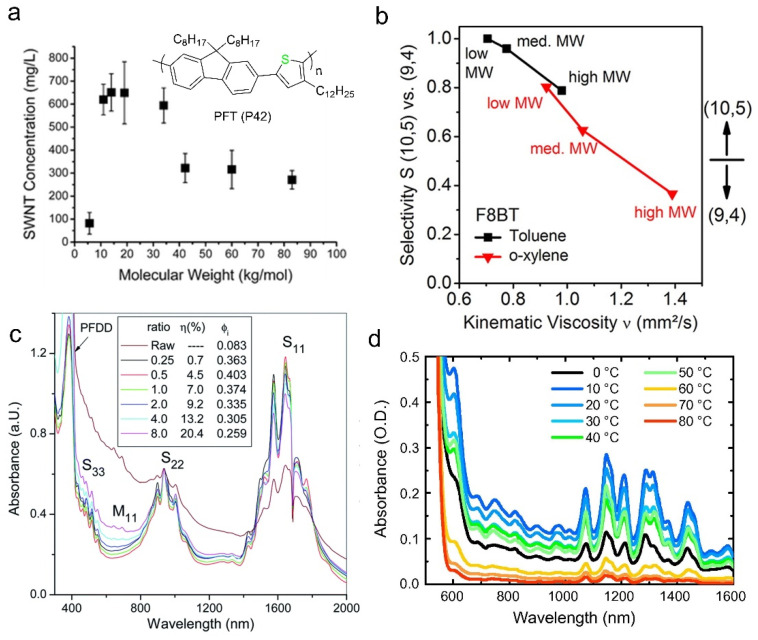
(**a**) The sorted-SWNT concentration changes with PFT molecular weight. The dispersion effect is the best when the molecular weight is between 10–35 kg/mol. Reproduced from Imin, P. et al., 2011; (**b**) the kinematic viscosity and selectivity vary with the polymer molecular weights and solvents. Higher molecular weights and o-xylene will bring higher viscosity and selectivity change. Reprinted with permission from Jakubka, F. et al., 2012. Copyright 2012 American Chemical Society; (**c**) the absorption spectra of the sorted solutions when using different PFDD/SWNT mass ratios (0.25–8). As the ratio increases, the sorted-SWNT concentration increases while the purity decreases. Reproduced from Ding, J. et al., 2014; (**d**) the absorption spectra of P3DDT/SWNT solutions prepared at different ultrasonic temperatures. The highest dispersion concentration was achieved at 10–20 °C. Reprinted with permission from Gomulya, W. et al., 2015. Copyright 2014 Elsevier Ltd.

In general, polymers could show good to excellent selectivity within a certain range of molecular weights [117,126]. If the molecular weight is too low or the polymer units are too few, the interaction between the polymer and the SWNT will be too weak to exfoliate or disperse SWNTs. Thus, the polymer will not exhibit its dispersion ability and selectivity [39,92]. If the molecular weight is too large, the solubility of the polymer may decrease, and the viscosity of the sorting solution will increase, which is detrimental to the selectivity [95,127]. Imin et al. synthesized a polymer, named PFTs, with molecular weights ranging from 5 to 85 kg/mol, they found that PFT/SWNT complexes had the best solubility when the molecular weights are between 10 to 35 kg/mol (Figure 10a) [126]. They thought that, with lower polymer weights, the oligomers could not wrap SWNTs effectively, due to their weak interactions with tubes. Therefore, the SWNTs easily bundled together and reaggregated during centrifugation. Mayor et al. got a similar result, finding that oligomers with low molecular weight are usually not sufficient to produce stable s-SWNTs dispersion when using PFDD as the sorting polymer [92]. However, when the molecular weights are high, the polymer PFT itself becomes not very soluble, it would aggregate with each other rather than wrapping around SWNTs [126]. Jakubka et al. used F8BT and PFO with molecular weights at different levels, low, medium and high, to purify s-SWNTs in toluene or o-xylene (Figure 10b) [127]. They found that the dispersion concentration increased, while the selectivity decreased when using high molecular weight polymers. They proposed that a higher viscosity of the sorted solution could stabilize more polymer/SWNT complexes, and prevented them from reaggregation and precipitation. Therefore, a higher yield while a reduced selectivity would be obtained.

### 3.3. Polymer/SWNT Ratio

The polymer/SWNT ratio used for sorting will also affect the purity and yield. Generally, a high polymer/SWNT ratio will result in an increased yield while a decreased purity. For a specific polymer, it is necessary to explore the appropriate polymer/SWNT ratio and solution concentration, to balance the yield and purity [78,79]. Ding et al. increased the mass ratio of PFDD/SWNT from 0.25 to 8, and they observed that the sorting yield improved from 0.7% to 20.4% [39]. Then, they used the ratio between 0.5–1.0 to achieve both high purity and high yield, as demonstrated by UV-Vis-NIR absorption spectrum (Figure 10c). Stuerzl et al. obtained the highest s-SWNTs purity when they adopted a polymer/SWNT ratio of 1/1 (*w*/*w*) (they used PFO, F8BT, and PFDD) and a solution concentration of 0.1~0.2 mg/mL [128]. For example, they extracted 90% (7, 6) SWNTs using 10/10 (mg/mg) PFDD/SWNT in 70 mL toluene. Using excess PFDD would disperse more other chiral SWNTs, such as (7, 5), (8, 6) and (8, 7) SWNTs. When the polymer/SWNT ratio is small, only the specific type of SWNTs could be well wrapped and dispersed. Other chiral SWNTs could not be effectively wrapped and, therefore, they easily aggregate and bundle together, settle as sediment after centrifugation. However, excess polymers disperse more types of SWNTs, resulting in a higher dispersion concentration with a lower purity. Furthermore, we can take multiple extractions to improve the sorting yield or taking second centrifugation to get higher purity [39,42].

### 3.4. Solvent

The solvent used for sorting has a great impact on the selectivity. The solubility of polymer and SWNTs, the conformation of polymer, and the interaction between polymer and SWNTs are closely related to the solvent. Many reports have studied the influence of solvents on sorting in terms of the solvent density, viscosity, dielectric constant/dipole moment and polarity [34,70,86,116,127,129]. The physical properties of solvents commonly used for separation are shown in Table 1. Overall, the polymer should be well dissolved in the solvent while the solubility of SWNTs should be not good. Only the SWNTs wrapped by the polymer can be well selected and dispersed. In addition, the density of the solvent should be lower than the SWNTs bundles (~1.3 g/cm^3^), ensuring the bundles can be precipitated during centrifugation.

Hwang et al. studied the effects of toluene, THF, chloroform and xylene on the selectivity of PFO and F8BT [86]. They found that, when using solvents which show better solubility for SWNTs, such as THF and chloroform, the polymer exhibits poor selectivity. Additionally, the density of chloroform is relatively large (1.5 g/cm^3^), it can stabilize SWNTs bundles and lead the bundles stay in the sorted solution. Cheng et al. found that the solvent viscosity would affect the stabilization of SWNTs dispersion [131]. However, they did not use conjugated polymers for dispersing SWNTs. Later Jakubka et al. argued that the viscosity of dispersed solution would influence the reaggregation rate of SWNTs, as well as the selectivity of the polymer (Figure 10b) [127]. As mentioned above, high viscosity solution could stabilize the slightly unstable polymer/SWNT complexes, leading to more SWNTs being dispersed. Therefore, the selectivity would also change, due to more metastable SWNT species being selected. Notice that the viscosity of the solution is closely related to the molecular weight and concentration of the polymer. Therefore, the effect of the solvent viscosity on the selectivity is not very clear.

Qian et al. used F8T2 to sort arc discharge SWNTs in solvent with different dipole moment (0–1.75 D) [70]. They found that the selectivity to s-SWNTs only occurred in m-xylene (0.32 D), toluene (0.36 D), and xylene (the mixture of m-xylene, o-xylene and p-xylene). Bao et al. compared six solvents (toluene, m-xylene, o-xylene, tetralin, decalin, and THF) with different polarities for sorting SWNTs by rr-P3DDT [116]. They found the s-SWNTs could be selectively dispersed in all non-polar solvents. They explained the selectivity through different polarizabilities of m-SWNTs and s-SWNTs. As we described in the mechanism section, the polar solvent will stabilize the dipole interactions of polymer/m-SWNT complexes and impede their reaggregation and precipitation. However, there are a few reports showing that s-SWNTs can be selectively dispersed in polar solvents. Therefore, the best solvent for sorting should be selected in combination with other factors, such as the type of polymers and the polymer weight.

### 3.5. Dispersion Temperature

Sorting temperature will affect the solubility and conformation of the polymer, as well as the interactions between the polymer and SWNTs, and eventually it will affect the sorting yield and purity. Bao et al. studied the sorting properties of rr-P3DDT at different temperatures [65]. They varied the temperatures from −40 °C to 90 °C, and found that as the temperature rose, the sorting yield increased first and then decreased with the maximum yield at 50 °C. They proposed a model in terms of thermodynamics and kinetic effects: (1) at high temperatures, the polymer is in a thermodynamically unstable state and the side chains become more flexible, which prevents the formation of stable polymer/SWNTs complexes and results in a low sorting yield; (2) at low temperatures, the solubility of the polymer decreases and the polymer backbone lacks conformational freedom to wrap around SWNTs. Therefore, the wrapping process will be limited by kinetics, leading to the low sorting yield again. Ultimately, only at medium temperatures can a high yield be obtained. Loi et al. also used P3DDT to study the effect of temperature on sorting. They found that the highest dispersion concentration could be obtained at temperatures between 10 °C to 20 °C (Figure 10d) [132]. Temperature ranges suitable for sorting may depend on different molecular weight or polymer/SWNT ratio. Chen et al. used PFH-A to extract (9, 8) SWNTs at different temperatures ranging from 0 °C to 100 °C. They found that the highest purity could be obtained at 0 °C [79]. Both the relative abundance and fluorescence intensity of (9, 8) SWNTs showed downward trends with increased temperature. They proposed that as the temperature raising, the stability of polymer/SWNT complex decreases, and polymers that originally wrapped around SWNTs will release from the surface of SWNTs, therefore, more SWNTs bundle together and participate. Han et al. also found that higher selectivity could be obtained at lower temperatures in non-polar solvents [133]. Overall, for a specific polymer, we need to consider its characteristic, such as molecular weight and solubility, and to find the best sorting temperature. When ensuring that the polymer is well dissolved, a higher selectivity can often be obtained at lower temperatures.

### 3.6. Other Factors

Except for the above parameters, the ultrasonic power and ultrasonic time, as well as centrifugal speed and centrifugal time should also be considered. The power and time used for sonication determine the exfoliation and dispersion levels. The choice of ultrasonic power depends on the specific instrument and needed ultrasonic time. Generally, the ultrasonic time is less than 2 h. A longer time will reduce the tube length and introduce more defects to SWNTs, which is harmful for their electrical applications, such as high performance SWNT FETs (usually 30 min ultrasonic time is used with adequate power) [36,134]. The centrifugal speed and time determine the settlement of the bundles and aggregations, which affects the purity of s-SWNTs. These two parameters also relate to the centrifugal apparatus used and the values are quite different in different reports. For example, Nish et al. could sort HiPCO SWNTs by F8BT centrifuging at 9000 g for 3 min [34]. Tange et al. sorted arc discharge SWNTs by F8BT centrifuging at 206,000 g for 2.5 h [48]. From our observation, higher centrifugal speed and longer centrifugal time usually lead to higher selectivity, but lower yield and throughput, which agrees well with the sorting mechanism discussed above.

## 4. Applications of Polymer-Sorted SWNTs

**Figure 11:**
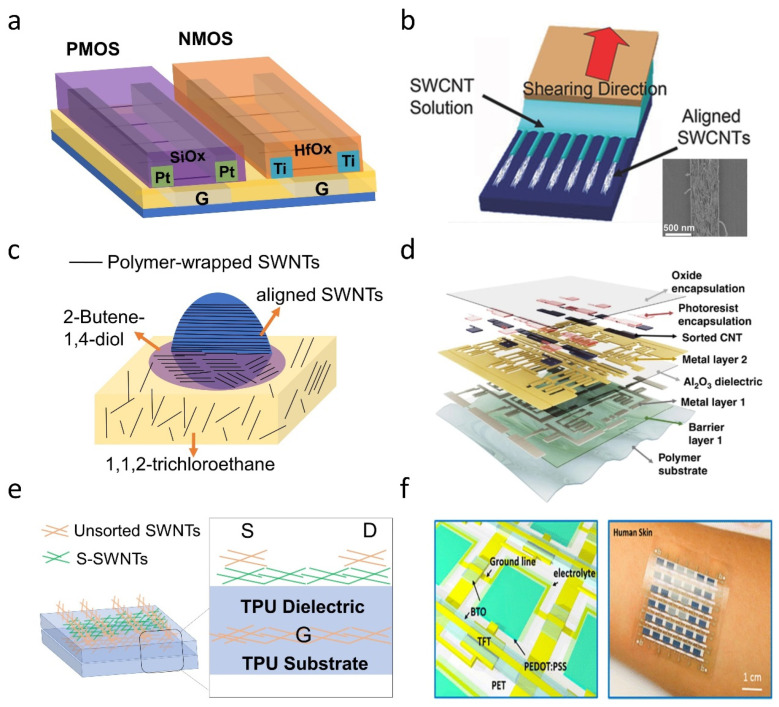
(**a**) Carbon nanotube (CNT)-based CMOS achieved by using dielectric doping (SiO_x_ for p-doping and HfO_x_ for n-doping) and metals with suitable work functions (Pt for p-type contacting and Ti for n-type contacting); (**b**) solution-shearing method for CNT alignment and the SEM image of the well-aligned SWNTs. Reprinted with permission from Park, S. et al., 2015. Copyright 2015 John Wiley and Sons; (**c**) dimension-limited self-alignment (DLSA) method for CNT alignment; (**d**) layer structure of high-performance flexible logic circuits operating at low voltage. Reprinted with permission from Lei, T. et al., 2019. Copyright 2019 Springer Nature; (**e**) stretchable and twistable SWNT transistor device based on polyurethane (TPU); (**f**) diagram of large-area and fully screen-printed active-matrix electrochromic displays (AMECD), based on flexible PET substrate and the photograph of that flexible AMECD sticking on human arm and displaying a picture of “U”. Reprinted with permission from Cao, X. et al., 2016. Copyright 2016 American Chemical Society.

s-SWNTs have shown great potential for electronic applications, due to their unique properties, such as nanometer scale, high charge carrier mobility, good mechanical stability, and solution processability. Compared with devices based on traditional semiconductors (e.g., Si), those fabricated by s-SWNTs can achieve smaller dimensions, lower power consumption, faster switching speed, as well as good flexibility and even stretchability, which represents the development of future electronic technology. It is worth noting that polymer-sorted s-SWNTs have been used in the preparation of high-performance logic and analog circuits, flexible and stretchable electronics, etc. (Figure 11).

It is theoretically predicted that, compared with Si transistors, SWNT transistors have more than twice the performance improvement and exhibit half the power consumption, and therefore have a 5–10 times energy efficiency improvement [22,135,136]. Experiments have proved the SWNT transistors outperform Si transistors in terms of subthreshold slope (SS) and transconductance [136,137]. This encourages people to continue to explore the application of SWNTs in logic and analog circuits. Recently, Shulaker et al. built a microprocessor based on solution-processed SWNTs, demonstrating the commercial prospects of SWNTs in large-scale integrated circuits [138]. Through reasonable circuit design using standard electronic design automation (EDA) tools, they reduced the demand for semiconducting SWNT purity of large-scale integrated circuits from 99.999999% to 99.99%. They coated a standard photoresist (polymethylglutarimide) on the deposited SWNT film, and then mechanically exfoliated it to remove most CNT aggregates that have an adverse effect on the device yield.

Low-power Complementary Metal Oxide Semiconductor (CMOS) logic circuits require both n-type and p-type transistors. However, transistors based on s-SWNTs will exhibit the characteristics of p-type, due to the contact with high-work-function metals and the doping of oxygen or water in the air [139,140]. To this end, n-type s-SWNTs can be prepared by using n-type dopants [141,142,143], employing low work function metal (such as Sc, Ti) as source and drain electrodes [136,138,144], or taking a ”doped” dielectric layer (such as Al_2_O_3_, HfO_2_) as an encapsulation [145]. Shulaker et al. used a “MIXED” strategy: doping with dielectric layer and contacting with low work function metal (Figure 11a), to improve the stability of SWNT CMOS transistors on a wafer-scale [138]. Adronov et al. measured both p-type and n-type FET characteristics using SWNTs sorted by a novel alternating copolymer consisting of fluorene and 2,5-dimethoxybenzene [146]. The maximum hole and electron mobilities could achieve 19 and 7 cm^2^/V·s respectively. Additionally, they used a self-assembled monolayer octyltrichlorosilane (OTS) to modify the dielectric surface, which could reduce the operational hysteresis and help to improve the hole and electron mobilities [73,146].

High-density aligned SWNTs are necessary for high-performance digital electronics, because they have reduced contact resistance and improved output current, and they can achieve highly linear signal amplification [147]. Arnold et al. reported a dose-controlled, floating evaporative self-assembly (DFES) method for the alignment of SWNTs [53,54,148]. They could achieve a density of ~50 SWNTs μm^−1^ [53]. Recently, Zhou et al. used the DFES method to deposit high density aligned SWNTs and fabricated radio-frequency (RF) CMOS devices with maximum operation frequencies over 100 GHz [147]. In order to drive the required current for logic circuits, the density of aligned SWNTs should be 100–200 SWNTs μm^−1^ (SWNT pitch is between 5–10 nm) [22,149]. Bao et al. developed a solution-shearing technique for the alignment of densely packed SWNTs over a large area (Figure 11b) [9]. They obtained a density of 150–200 SWNTs μm^−1^, and a current density of 10.08 μA/μm at *V_DS_* = −1 V, which is 45 times higher than that of the random SWNT network. Recently, Peng et al. achieved a wafer-scale alignment of SWNTs using a dimension-limited self-alignment (DLSA) method (Figure 11c) [150]. They were able to prepare SWNT arrays with a density of 100–200 SWNTs μm^−1^ by adjusting the concentration of the carbon tube solution. The results of polarized Raman spectroscopy indicate that the angle distribution of the SWNT arrangement is on average 8.3°. They fabricated top-gate transistors using the aligned SWNTs and found that the peak transconductance reached 0.9 mS/μm, which is superior to all SWNT transistors reported previously.

High-performance flexible logic circuits have also been prepared. Tang et al. used an electrostatically doped method to fabricate SWNT-based flexible CMOS devices on polyimide substrate [55]. They deposited Al doped Al_2_O_3_ over the channel of FETs to n-dope SWNTs, and combining with original p-type FETs, they successfully fabricated flexible ring oscillators with a stage delay of only 5.7 ns. Bao et al. reported high-performance flexible logic circuits operating at low voltage of 3 V (Figure 11d) [42]. They built 5-stage ring oscillators and 8-stage shift registers on polyimide substrate by depositing s-SWNTs with a purity higher than 99.997%, and using a pseudo-CMOS design (using only unipolar p-type FETs). The ring oscillators worked with gate delays of 42.7 ± 13.1 ns, and the shift registers could run at 50 kHz with the first tunable-gain amplifier of 1000 gain at 20 kHz. These results are encouraging for fabricating large-scale flexible electronics with polymer-sorted SWNTs.

The polymer-sorted SWNTs can also be used for the preparation of flexible and stretchable devices. The solution-processed SWNTs network can exhibit good electrical performance with high mechanical flexibility and stretchability. It may replace or supplement traditional semiconducting materials in high-performance and low-cost thin-film transistor (TFT) devices, being used in display, sensing and wearable devices [151,152,153,154]. Bao et al. fabricated stretchable TFTs with polymer-sorted SWNTs as channel materials and unsorted SWNTs as electrodes (Figure 11e) [151]. They used polyurethane (TPU), which would prevent the tear propagation as dielectric and substrate. The device could withstand 100% strain and maintain its performance after 1000 stretching cycles, or even get impacted, punctured and torn. Joo et al. reported bendable and stretchable TFTs with aligned SWNT film which is deposited by an inverse dose-controlled, floating evaporative self-assembly (i-DFES) method [155]. They used ion-gel as the gate dielectric, and the ion-gel-based TFTs could bear 5% stretching and 10% bending without performance degradation. Zhou et al. reported fully printed and large area flexible active-matrix backplanes based on SWNT TFTs [152]. They then built active-matrix electrochromic displays (AMECD) with the integration of electrochromic pixels (Figure 11f). The device performed good switching characteristics and remained stable for a long time in the air (having negligible electrical performance degradation after 7 days), showing great potential for the large-scale manufacture of low-cost flexible displays.

## 5. Conclusions

To conclude, we have summarized recent progress on conjugated polymers used for selectively dispersing s-SWNTs and highlight the effect of polymer structures on the dispersion selectivity and yield. We then discussed possible mechanisms for sorting s-SWNTs, based on the polarizabilities of SWNTs and the electronic structures of polymers. Except for changing the polymer structures, we can also adjust the solvent, the polymer molecular weight, the polymer/SWNT ratio, as well as the ultrasonic and centrifugal conditions, to achieve both high selectivity and high yield. Although polymer-sorted SWNTs have been used in the preparation of high-performance logic/analog circuits, and flexible/stretchable electronics, there are still some problems that need to be addressed before their practical application. First of all, it is necessary to further improve the purity and sorting yield of s-SWNTs, and it is better to achieve the enrichment of SWNTs with specific chirality or small diameter distribution, to ensure the uniformity of the electrical performance for high-performance devices. Second, it is desired to further explore the solution processing methods for the dense alignment of SWNTs, especially for high-performance computing. Third, most of the polymers wrapped on SWNTs cannot be removed completely; thus, how to eliminate the impact of polymers on the device performance needs expanded research. The settlement of these problems also points out a direction that might be useful for the future development of the conjugated polymer sorting method.

## Figures and Tables

**Table 1:** Physical properties of commonly used solvents for sorting SWNTs [70,130].

Solvent	Density (g/mL)	Viscosity (mPa.s)	Topological Polar Surface Area (Å²)	Dielectric Constant	Dipole Moment (Hexane 0 D)
Toluene	0.87	0.56	0	2.376	0.36D
*p*-xylene	0.86	0.603	0	2.27	0.07D
*m*-xylene	0.86	0.581	0	2.367	0.32D
*o*-xylene	0.88	0.76	0	2.568	0.54D
THF	0.89	0.50	9.2	7.4	1.75D
chloroform	1.48	0.556	0	4.65	1.04D
